# Hidden in plain sight: novel molecular data reveal unexpected genetic diversity among paramphistome parasites (Digenea: Paramphistomoidea) of European water frogs

**DOI:** 10.1017/S003118202200083X

**Published:** 2022-09

**Authors:** Michal Benovics, Peter Mikulíček, Zuzana Žákovicová, Petr Papežík, Camila Pantoja

**Affiliations:** 1 Department of Botany and Zoology, Faculty of Science, Masaryk University, Kotlářská 2, 611 37 Brno, Czech Republic; 2 Department of Zoology, Faculty of Natural Sciences, Comenius University in Bratislava, Ilkovičova 6, 842 15 Bratislava, Slovakia; 3 Institute of Ecology, Nature Research Centre, Akademijos 2, 08412 Vilnius, Lithuania;; 4 Institute of Parasitology, Biology Centre of the Czech Academy of Sciences, Branišovská 31, 370 05 České Budějovice, Czech Republic

**Keywords:** Balkan Peninsula, *Diplodiscus subclavatus*, haplotype diversity, *Opisthodiscus diplodiscoides*, *Pelophylax*, population genetics

## Abstract

Parasites might represent a helpful tool in understanding the historical dispersion and phylogeography of their hosts. In order to reveal whether the migration routes and diversification of hosts can be traceable in the genetic structures of their parasites, we investigated the diversity of paramphistomoid trematodes of *Pelophylax* frogs in 2 geographically distant European regions. Water frogs belonging to the genus *Pelophylax* represent a striking example of a species with a high variety of ecological adaptations and a rich evolutionary history. The parasites were collected from 2 Balkan endemic species, *P. epeiroticus* and *P. kurtmuelleri*, and 2 species in Slovakia, *P. esculentus* and *P. ridibundus*. While in Slovakia, *Pelophylax* frogs harboured 2 species, the diplodiscid *Diplodiscus subclavatus* and the cladorchiid *Opisthodiscus diplodiscoides*, only the former was recorded in the south-western Balkans. Remarkably high genetic diversity (16 unique mitochondrial *cox*1 haplotypes, recognized among 60 novel sequences) was observed in *D. subclavatus*, and subsequent phylogenetic analyses revealed a strong population-genetic structure associated with geographical distribution. We also evidenced the existence of 2 divergent *D. subclavatus cox*1 haplogroups in the south-western Balkans, which might be associated with the historical diversification of endemic water frogs in the regional glacial microrefugia.

## Graphical abstract

**Figure:**
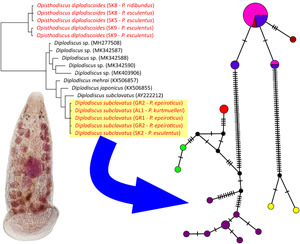

## Introduction

Gradually decreasing amphibian diversity is generally viewed as an early-warning signal of problems in the environment (Niemi and McDonald, 2004). Nonetheless, the decrease is often likely due to a combination of a variety of factors, of which one of the most notable is the effect of parasitic organisms (Blaustein and Wake, 1995; Semlitsch, 2003). Amphibians inhabit a multitude of habitats and exhibit a striking diversity of life history patterns, reproductive modes, body sizes and trophic relations (Wells, 2007). Due to their physiological dependence on the aquatic environment, they represent optimal hosts for parasites, since the development and transmission of the infective stages of many parasites takes place in the same aquatic environment. This places amphibians into the role of an interesting model for studying parasite diversity, especially in regions considered as the world's biodiversity hotspots. Nonetheless, they are still one of the least explored groups in this regard (Aho, 1990; Barton, 1999; Poulin and Morand, 2000; Carlson *et al*., 2020).

Due to their wide occurrence and unique reproduction mode, some of the best-studied amphibians in Europe are water frogs of the genus *Pelophylax*. Currently, at least 14 species and 3 asexual hybrid forms live in the western Palearctic (Plötner, 2005; Frost, 2021). The distribution of water frog species is unequal and putatively intimately linked with both historical, geological and climatic processes and geographic heterogeneity in the region (Beerli *et al*., 1996; Lymberakis *et al*., 2007; Akın *et al*., 2010; Plötner *et al*., 2010). Remarkable species diversity and high endemism are observed in the peri-Mediterranean area, especially in the Balkans and the Middle East (Speybroeck *et al*., 2016; Dufresnes *et al*., 2017). To the north, species diversity is declining, with only 2 species living in central and northern Europe – specifically, *P. ridibundus* (Pallas, 1771) and *P. lessonae* (Camerano, 1882) – whose extensive hybridization gave rise to an asexual hybrid form, *P. esculentus* (Linnaeus, 1758) (Graf and Polls Pelaz, 1989; Plötner, 2005). According to molecular data, basal radiation among the major European *Pelophylax* lineages took place in the middle Miocene, with subsequent species diversification during the upper Miocene and the Pliocene (Beerli *et al*., 1996; Lymberakis *et al*., 2007; Akın *et al*., 2010). The distribution and current intraspecific diversity of water frogs were also influenced by Pleistocene climatic oscillations, which led to the retraction of populations to the climatically favourable southern refugia during the glacials, and later expansion to the north in interglacials (Snell *et al*., 2005; Hoffmann *et al*., 2015).

Although the current distribution, phylogeny and genetic diversity of *Pelophylax* species are relatively well known, this is no longer the case with their parasites. Water frogs are important hosts for many parasitic groups, from protists to arthropods (e.g., Günther, 1990; Herczeg *et al*., 2016; Chikhlyaev *et al*., 2018; Iacob, 2021). One of the common parasites of amphibians are paramphistomoid trematodes of the families Diplodiscidae Cohn, 1904 and Cladorchiidae Fischoeder, 1901, which have been reported from frogs all around the world (e.g., Skrjabin, 1949; Yamaguti, 1971; Sey, 1991; Vojtková and Roca, 1994; Jones, 2005). Since the original descriptions of the families, taxonomic revisions have included several reorganizations of genera and formerly described subfamilies according to their morphological traits and/or geographical distribution (e.g., Cohn, 1904; Skrjabin, 1949; Odening, 1959; Yamaguti, 1971; Grabda-Kazubska, 1980; Sey, 1983). Diplodiscidae and Cladorchiidae share several morphological characters (e.g., the absence of an oral sucker; a sucker-like attachment organ at the posterior end; paired pharyngeal sacs; and an absent pharyngeal bulb) and can be differentiated primarily by the number of testes – representatives of Diplodiscidae have 1, while Cladorchiidae have 2 (Jones, 2005; Sey, 2005). Moreover, these 2 families exhibit highly different host ranges; while Diplodiscidae include only a few genera, predominantly parasitizing in amphibians and reptiles, numerous highly diversified Cladorchiidae genera can be found in the guts of various marine and freshwater fishes, amphibians, reptiles and mammals (Jones, 2005).

So far, only 1 Diplodiscidae species is reported from European water frogs. *Diplodiscus subclavatus* (Pallas, 1760) is a common species which parasitizes the rectum of amphibians all across Europe, with a distribution range from the Iberian Peninsula in the west up to the Ukraine and Turkey in the East (e.g., Bailenger and Chanseau, 1954; Gomez, 1987; Vojtková and Roca, 1994; Navarro and Lluch, 2006; Bjelić-Čabrilo *et al*., 2009; Popiołek *et al*., 2011; Düşen and Öz, 2013; Karakaş, 2015; Herczeg *et al*., 2016; Kuzmin *et al*., 2020; Čeirāns *et al*., 2021). The family Cladorchiidae is represented by 2 species parasitizing water frogs in Europe – *Opisthodiscus diplodiscoides* Cohn, 1904 and *O. nigrivasis* Mehély, 1929. Species of this genus are less prevalent and are reported from France (Dollfus, 1951; Bailenger and Chanseau, 1954; Combes *et al*., 1973), Germany (Andreas, 2007), Slovakia (Kopřiva, 1957; Vojtková, 1974), Poland (Grabda-Kazubska, 1980), Spain (Combes and Gerbaux, 1970; Combes and Sarrouy, 1971; Martínez-Fernández *et al*., 1988) and Hungary (Mehély, 1929; Sey, 1991). The 2 *Opisthodiscus* species have often been considered a single taxon, since their morphological differentiation is not fully clear (Odening, 1959), and, therefore, their hosts and expected distribution have to be viewed with caution.

The distribution and diversity of paramphistome parasites of frogs were investigated to some degree in the past; however, little is known about their genetic diversity in this group of hosts. Publicly available sequence data on diplodiscid parasites of frogs are limited and include only several partial sequences of genes coding ribosomal subunits for 2 *Diplodiscus* species (*D. mehrai* Pande, 1937 and *D. japonicus* (Yamaguti, 1936) from Besprozvannykh *et al*., 2018) and 1 species of *Catadiscus* (*C. marinholutzi* Freitas & Lent, 1939 from Queiroz *et al*., 2021). To date, there is no molecular data for any species of *Opisthodiscus*, which could be used to assess phylogenetic relationships based on DNA sequences. Although numerous partial *18S* and *28S* rRNA sequences from *Diplodiscus* specimens, with unambiguous species designation, and from *D. amphichrus* (Tubangi, 1933) originating from Indian *Euphlyctis cyanophlyctis* (Schneider, 1799) are available, they have not been used in any previous publication and are, therefore, considered unreliable. In addition, only Besprozvannykh *et al*. (2018) and Queiroz *et al*. (2021) investigated more thoroughly the phylogenetic relationships among diplodiscids using available genetic markers; however, neither study fully tackled the intraspecific variability in the respective species. Regarding mitochondrial DNA (mtDNA), the complete mitogenome of *D. nigromaculati Wang*, 1977 from the east Asian water frog species *P. nigromaculatus* (Hallowell, 1861) was recently added to public databases; however, these data have not yet been utilized in any published work.

The present study therefore aimed to reveal the distribution, prevalence and abundance of paramphistomes in European water frogs in 2 geographically distant regions (central Europe and the south-west Balkans), and to assess the intraspecific genetic variability among collected paramphistomes using highly variable genetic markers, i.e., mtDNA genes – a novel method in the study of Diplodiscidae. On the basis of knowledge about amphibian hosts, we can hypothesize that (1) endemic species will be parasitized by endemic paramphistome taxa with a limited distribution range, and (2) the genetic diversity of recent parasite lineages and the relationships among them will be intimately linked with the evolutionary history and historical dispersion patterns proposed for European water frogs. Furthermore, we also assume that (3) *Pelophylax* hybrid forms might share parasite lineages associated with parental species in the region of sympatric occurrence (e.g., in the Slovakia – *P. esculentus* × *P. ridibundus*).

## Material and methods

### Material collection, parasite fixation and identification

**Fig. 1.**
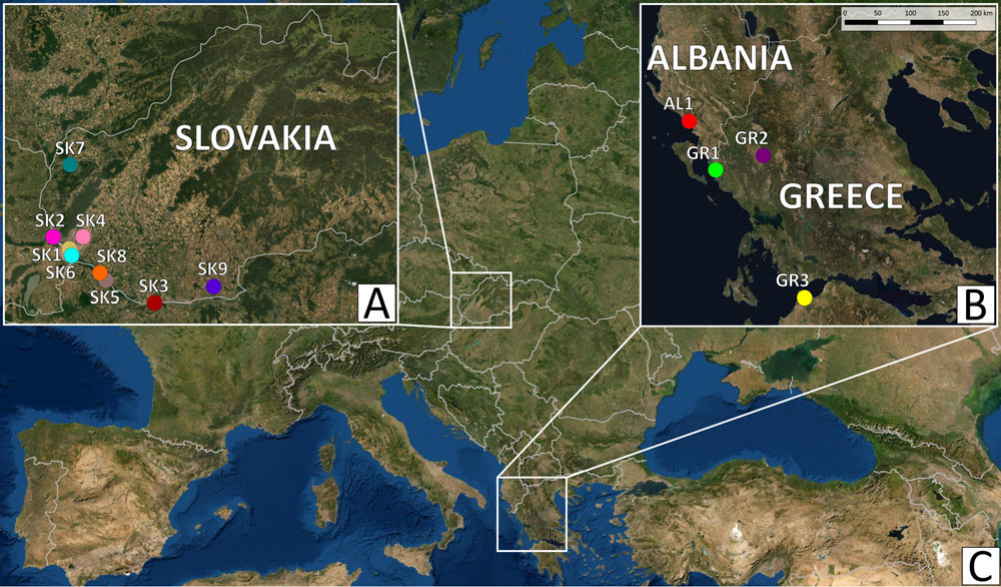
Map showing the collection sites in Slovakia and the Balkans. A, the collection sites in the Slovakia; B, the collection sites in the Balkans; C, map of the Europe. The same scale bar is applicable for parts A and B. The same colouration palette of the collection site points is used for the sites in the subsequent figures.

A total of 161 *Pelophylax* water frogs from 13 localities in the Balkans and Slovakia were examined for the presence of parasites (Fig. 1, Table 1) Trematodes were collected from 4 host species: *P. esculentus* (*n* = 50) and *P. ridibundus* (*n* = 72) in Slovakia, and *P. epeiroticus* (Schneider, Sofianidou & Kyriakopolou-Sklavounou, 1984) (*n* = 30) and *P. kurtmuelleri* (Gayda, 1940) (*n* = 9) in Greece and Albania, respectively. Although the latter 2 species have been considered as endemics of the Balkan Peninsula since description, haplotypes and alleles of *P. kurtmuelleri* have been recorded in several European countries in the last few decades (e.g., Dufresnes *et al*., 2017, 2018; Herczeg *et al*., 2017; Kolenda *et al*., 2017; Bellati *et al*., 2019 and literature therein; Litvinchuk *et al*., 2020). Water frog species were identified according to morphological traits (Günther, 1990; Plötner, 2005) and molecular markers – specifically, sequences of the mitochondrial *ND2* fragment and microsatellites. Details of *ND2* and microsatellite laboratory analyses and the genetic identification of water frogs have been studied by Plötner *et al*. (2008), Hoffmann *et al*. (2015) and Papežík *et al*. (2021).

**Table 1.** Quantitative descriptors of the populations of collected paramphistomes calculated for each metapopulation

Country	LocID	Collection site name	Coordinates	Host	NH	*Diplodiscus*				*Opisthodiscus*			
						N	*P*	A	I	N	*P*	A	I
Slovakia	SK1	Chorvátske rameno	48°05′59.50″N 17°07′46.90″E	*P. ridibundus*	10	–	–	–	–	1	10%	0.10 (0.00–0.30)	1
	SK2	Devín	48°10′29.80″N 16°58′36.10″E	*P. esculentus*	10	17	80%	1.70 (0.90–2.50)	1–4	–	–	–	–
				*P. ridibundus*	11	29	64%	2.64 (1.27–4.73)	1–9	–	–	–	–
	SK3	Gbelce	47°51′41.61″N 18°31′17.43″E	*P. ridibundus*	11	7	18%	0.64 (0.00–1.82)	2–5	–	–	–	–
	SK4	Ivánske jazero	48°10′33.50″N 17°15′45.20″E	*P. ridibundus*	10	–	–	–	–	4	40%	0.40 (0.00–0.60)	1
	SK5	Kráľovská Lúka	47°53′54.20″N 17°28′53.90″E	*P. ridibundus*	10	–	–	–	–	4	40%	0.40 (0.10–0.60)	1
	SK6	Rusovce	48°03′26.80″N 17°09′11.20″E	*P. esculentus*	10	–	–	–	–	9	60%	0.90 (0.40–1.50)	1–3
	SK7	Šaštín-Stráže	48°37′55.70″N 17°08′21.70″E	*P. esculentus*	10	–	–	–	–	5	30%	0.50 (0.00–1.00)	1–2
	SK8	Šulianske jazero	47°56′46.20″N 17°25′23.60″E	*P. ridibundus*	10	–	–	–	–	9	60%	0.90 (0.30–1.30)	1–2
	SK9	Veľký Lél	47°45′36.76″N 17°56′40.24″E	*P. esculentus*	20	14	25%	0.70 (0.20–1.40)	2–5	11	35%	0.55 (0.23–0.90)	1–2
				*P. ridibundus*	10	25	40%	2.50 (0.40–6.74)	1–15	–	–	–	–
Albania	AL1	Qazim Pali	40°02′58.80″N 19°50′31.20″E	*P. kurtmuelleri*	9	19	33%	2.11 (0.22–5.22)	1–11	–	–	–	–
Greece	GR1	Igoumenitsa	39°32′11.70″N 20°12′13.70″E	*P. epeiroticus*	10	12	40%	1.20 (0.30–3.40)	1–7	–	–	–	–
	GR2	Ioannina	39°41′19.10″N 20°51′30.20″E	*P. epeiroticus*	10	38	70%	3.80 (1.60–7.75)	1–14	–	–	–	–
	GR3	Limanaki	38°10′21.90″N 21°25′18.70″E	*P. epeiroticus*	10	2	10%	0.20 (0.00–0.60)	2	–	–	–	–

NH, number of processed host individuals; *N*, number of collected parasite individuals; *P*, prevalence; *A*, mean abundance (a 95% confidence interval); *I*, range of intensity of infection; absence of data is marked by dashes (–).

A total of 206 paramphistomoid trematodes were collected from frog rectums and urinary bladders. First, all collected specimens were doused in heated physiological solution to ensure their flexibility. Then, preliminary morphological identification was carried out using an Olympus SZX7 stereomicroscope (Olympus, Tokyo, Japan). The specimens of each preliminarily identified parasite taxon were divided into 2 groups: 1–3 whole specimens were preserved in 96% ethanol for later DNA extraction, while the rest were used for morphological analyses. Prior to mounting in Canada balsam, these latter specimens were stained with iron-acetocarmine, following the protocol of Georgiev *et al*. (1986). Determination followed Jones (2005) and Vojtková (1974). Basic quantitative descriptors of the parasite populations, i.e., prevalence, mean abundance and minimum and maximum intensities of infection, were calculated for each parasite species according to Bush *et al*. (1997). Prevalence, as the percentage of frogs infected by a given parasite species, and mean abundance, as the mean number of parasite specimens per individual host taking into account both infected and uninfected hosts, were calculated. Following the suggestion of Rózsa *et al*. (2000) for interpreting quantitative data, a 95% confidence interval was calculated for mean abundance. Due to the relatively low host sample size per population, the bias-corrected and accelerated bootstrap (BCa) method QPweb (Reiczigel *et al*., 2019) was used to calculate a confidence interval for mean abundances.

### DNA extraction, amplification and sequencing

Genomic DNA was extracted following the protocol described by Georgieva *et al*. (2013). The partial region of *28S* rRNA (hereinafter abbreviated as *28S*) was amplified using the primers digl2 (5-AAG CAT ATC ACT AAG CGG-3) (Tkach *et al*., 2001) and 1500R (5-GCT ATC CTG AGG GAA ACT TCG-3) (Tkach *et al*., 2003); the additional internal primers ECD2 (5ʹ-CCT TGG TCC GTG TTT CAA GAC GGG-3ʹ) (Littlewood *et al*., 1997) and 300F (5ʹ-CAA GTA CCG TGA GGG AAA GTT G-3ʹ) (Littlewood *et al*., 2000) were used for Sanger sequencing. The partial mtDNA gene coding cytochrome *c* oxidase subunit I (hereinafter abbreviated as *cox*1) was amplified using the primers JB3 (forward; 5′-TTT TTT GGG CAT CCT GAG GTT TAT-3′) (Bowles *et al*., 1995) and CO1-R trema (reverse; 5′-CAA CAA ATC ATG ATG CAA AAG G-3′) (Miura *et al*., 2005). PCR reactions were performed in a total volume of 25 *μ*L and included 12.5 *μ*L of *MyFi™* Mix, 1.25 *μ*L of each primer (10 mm), 8 *μ*L of ddH_2_O and 1.5 *μ*L of genomic DNA. The cycling parameters of PCR amplification followed those of Cutmore *et al*. (2013) for *28S* and Koehler *et al*. (2011) for *cox*1. Amplified products were purified with Exo-SAP-IT Kit™ Express Reagent (Thermo Fisher Scientific Baltics UAB, Vilnius, Lithuania) following the manufacturer's instructions, and sequenced with the same primers as were used in PCR, or alternatively with internal primers. The ABI PRISM BigDye Terminator Cycle Sequencing Ready Reaction Kit (Applied Biosystems-Perkin Elmer, Waltham, Massachusetts) and the MegaBACE sequencer (GE Healthcare Life Sciences) were used for sequencing. Contiguous sequences were assembled using Geneious v. 11 (Biomatters, Auckland, New Zealand) and deposited in GenBank.

### Phylogenetic analyses

Two alignments (for *28S* and *cox*1) including novel sequences of paramphistomoids and orthologue sequences retrieved from GenBank were built using the fast Fourier transform algorithm implemented in MAFFT software (Katoh *et al*., 2002). The initial alignment built from orthologue sequences of *28S* was used to assess the taxonomical designation and phylogenetic position of the collected paramphistomoid trematodes. The dataset included 8 sequences of *Diplodiscus* spp. available in GenBank and 1 of *Catadiscus marinholutzi* (Diplodiscidae). To assess the phylogenetic relationships of the investigated trematodes to cladorchiids, homologue sequences of 15 cladorchiid species belonging to 10 genera were also included in the analyses. Following the phylogeny proposed by Alves *et al*. (2020), an outgroup comprising *Carmyerius spatiotus* (Brandes, 1898) (JX518958), *Fischoederius elongatus* (Poirier, 1883) Stiles & Goldberger, 1910 (JX518966), *Gastrothylax crumenifer* (Creplin, 1847) (JX518971) (Gastrothylacidae) and *Paramphistomum cervi* (Zeder, 1790) Fischoeder, 1901 (KJ459936) (Paramphistomatidae) was used for the rooting of the phylogram. The final alignment was manually trimmed to the shortest available sequence to unify the length. The general time-reversible evolutionary model (GTR) was applied for the subsequent phylogenetic analyses. The gamma shape and proportion of invariable sites were calculated with jModelTest 2.1.2 (Darriba *et al*., 2012). Phylogenetic trees were constructed using the Bayesian inference (BI) and maximum likelihood (ML) approaches in MrBayes 3.2 (Ronquist *et al*., 2012) and RaxML 8.1.12 (Stamatakis, 2006, 2014), respectively. BI analysis used the Metropolis-coupled Markov chain Monte Carlo algorithm with 2 parallel runs of 1 cold and 3 hot chains, which was run for 10^7^ generations, sampling trees every 100 generations. The initial 30% of all saved trees were discarded as ‘burn-in’ after checking that the standard deviation split frequency fell below 0.01. Whether the runs and parameters of individual runs converged was checked using Tracer 1.7.1 (Rambaut *et al*., 2018). Posterior probabilities (PP) for each tree node were calculated as the frequency of samples recovering a given clade. The clade bootstrap support (BS) for ML trees was assessed by simulating 10^3^ pseudoreplicates.

**Fig. 2.**
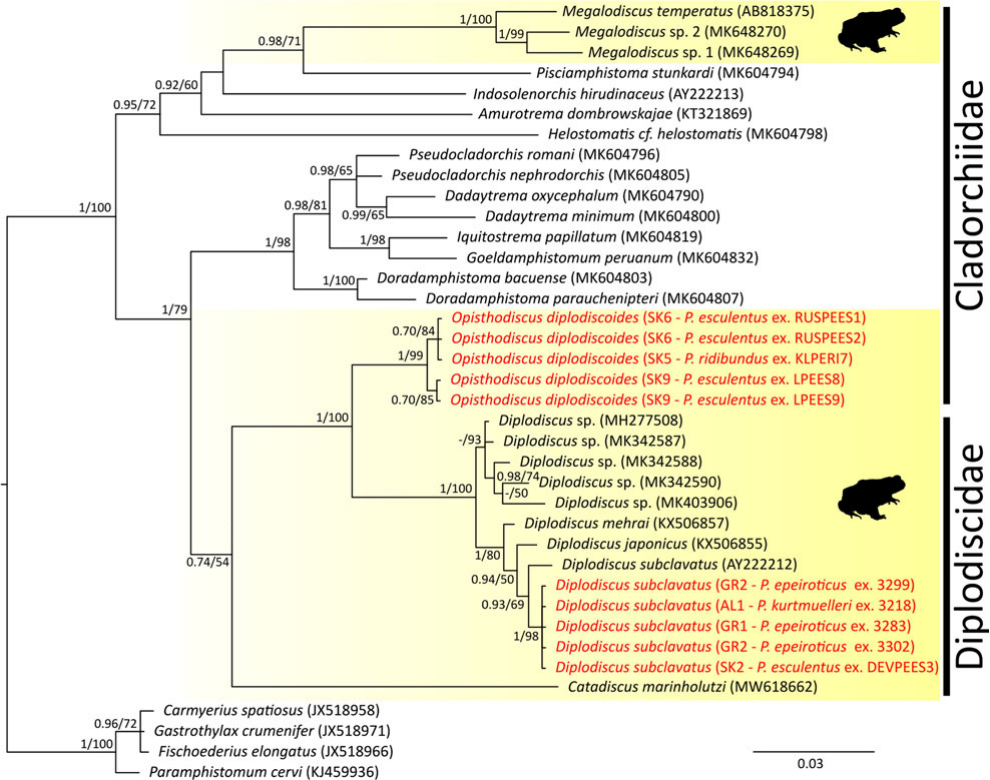
Phylogenetic tree of 25 *28S* sequences of 9 Paramphistomoidea species reconstructed by Bayesian inference. The tree is based on 810 bp-long sequences and rooted using *Carmyerius spatiosus, Gastrothylax crumenifer, Fischoederius elongatus* and *Paramphistomum cervi* as the outgroup. Values at the nodes indicate posterior probabilities (>70) from the Bayesian inference, and bootstrap values (>50) from the maximum likelihood analysis. Lower values are shown as dashes (–). The length of branches represents the number of substitutions per site. The silhouettes at the taxa with coloured background represent common hosts of the respective trematode species – frogs. The new sequences generated from this study are in red.

**Fig. 3.**
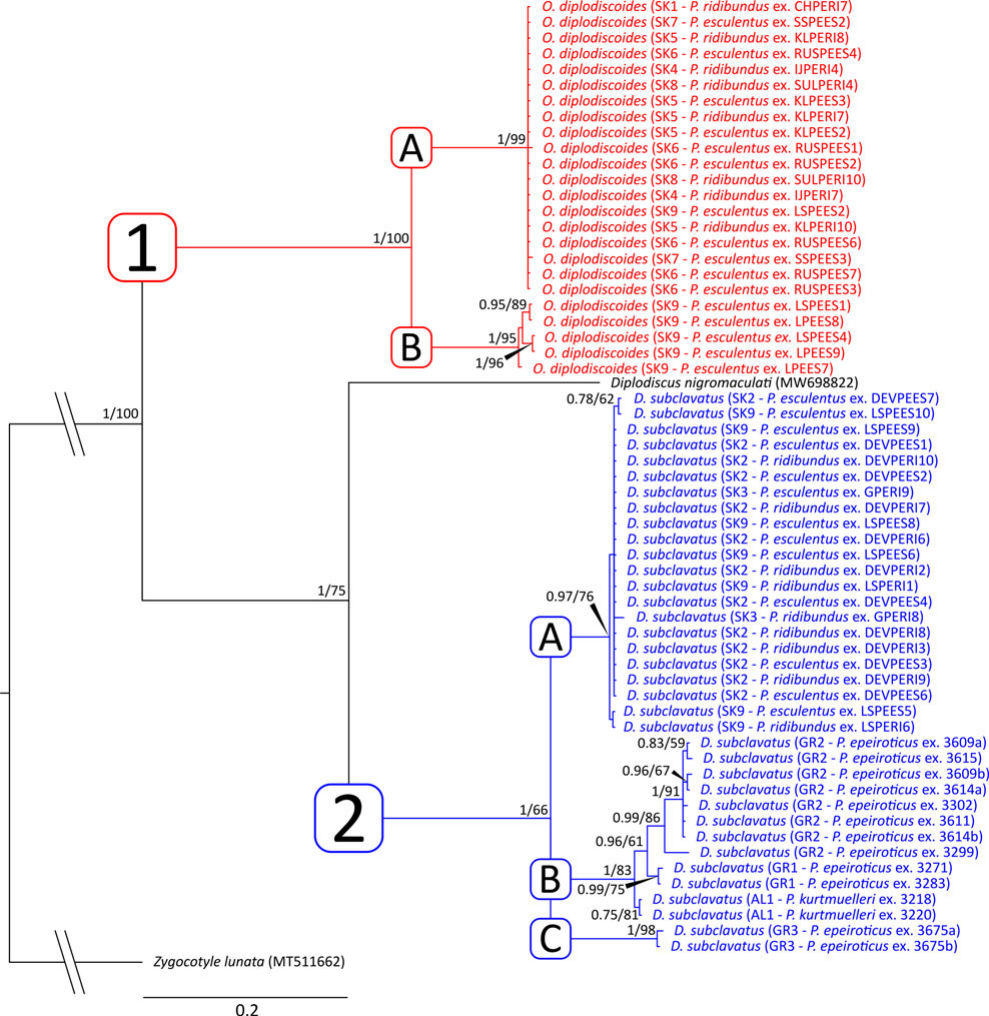
Phylogenetic tree of 61 *cox*1 sequences of 3 Paramphistomoidea species reconstructed by Bayesian inference. The tree is based on 683 bp-long sequences and rooted using *Zygocotyle lunata* as the outgroup. Values at the nodes indicate posterior probabilities (>70) from the Bayesian inference, and bootstrap values (>50) from the maximum likelihood analysis. Lower values are shown as dashes (–). The length of branches represents the number of substitutions per site. The numbers and letters in the squares represent clades and lineages further discussed in the Results section. The new sequences generated from this study are shown in colour: red – *Opisthodiscus diplodiscoides*; blue – *Diplodiscus subclavatus*.

The alignment built of *cox*1 sequences was used to assess the genetic diversity among the collected paramphistomoids and included 60 newly generated sequences and an orthologue sequence of *D. nigromaculati* extracted from its complete mitochondrial genome data (accession number MW698822). All sequences were trimmed to equalize their length and translated into amino acids to avoid any signal misreads. The sequence data were treated as codon partitioned, and a GTR model was selected independently for each position within the codon, including both a gamma distribution and the proportion of invariable sites. ML and BI analyses were conducted with the same number of iterations as for *28S*. The *cox*1 sequence of *Zygocotyle lunata* (Diesing, 1836) (MT511682) (Paramphistomatidae) was used as the outgroup for rooting the phylogram. The level of DNA polymorphism in *cox*1 sequences, i.e., haplotype diversity (*Hd*), nucleotide diversity (*π*), number of unique haplotypes and number of variable sites, was assessed using DnaSP 5 (Librado and Rozas, 2009). The median-joining haplotype networks, constructed in PopART (Leigh and Bryant, 2015) for each species individually, were used to assess population-genetic structure based on *cox*1 haplotypes. All new sequences generated in the present study are presented in Table 2, and those downloaded from GenBank are presented in Figs 2 and 3.

**Table 2.** A list of sequenced *Diplodiscus subclavatus* and *Opisthodiscus diplodiscoides* individuals with respective hosts and collection sites

Species	Country	Collection site (LocID)	ID	Haplotype	Host	Acc. num. *28S*	Acc. num. *cox*1
*Diplodiscus subclavatus*	Albania	Qazim Pali (AL1)	3218*	D_H7	*P. kurtmuelleri*	ON660887	ON647344
			3220	D_H7	*P. kurtmuelleri*	–	ON647345
	Greece	Igoumenitsa (GR1)	3271	D_H9	*P*. *epeiroticus*	–	ON647346
			3283*	D_H8	*P*. *epeiroticus*	ON660888	ON647347
		Ioannina (GR2)	3299*	D_H10	*P*. *epeiroticus*	ON660889	ON647348
			3302*	D_H16	*P*. *epeiroticus*	ON660886	ON647349
			3609a	D_H11	*P. epeiroticus*	–	ON647350
			3609b	D_H14	*P. epeiroticus*	–	ON647351
			3611	D_H12	*P. epeiroticus*	–	ON647352
			3614a	D_H13	*P. epeiroticus*	–	ON647353
			3614b	D_H12	*P. epeiroticus*	–	ON647354
			3615	D_H15	*P. epeiroticus*	–	ON647355
		Limanaki (GR3)	3675a	D_H5	*P. epeiroticus*	–	ON647356
			3675b	D_H6	*P. epeiroticus*	–	ON647357
	Slovakia	Devín (SK2)	DEVPEES3*	D_H1	*P*. *esculentus*	ON660895	ON647361
			DEVPEES1	D_H1	*P. esculentus*	–	ON647359
			DEVPEES2	D_H1	*P. esculentus*	–	ON647360
			DEVPEES4	D_H1	*P*. *esculentus*	–	ON647362
			DEVPEES6	D_H1	*P*. *esculentus*	–	ON647363
			DEVPEES7	D_H3	*P. esculentus*	–	ON647364
			DEVPERI2	D_H1	*P. ridibundus*	–	ON647366
			DEVPERI3	D_H1	*P*. *ridibundus*	–	ON647367
			DEVPERI6	D_H1	*P*. *ridibundus*	–	ON647368
			DEVPERI7	D_H1	*P*. *ridibundus*	–	ON647369
			DEVPERI8	D_H1	*P*. *ridibundus*	–	ON647370
			DEVPERI9	D_H1	*P*. *ridibundus*	–	ON647371
			DEVPERI10	D_H1	*P. ridibundus*	–	ON647365
		Gbelce (SK3)	GPERI8	D_H2	*P*. *ridibundus*	–	ON647372
			GPERI9	D_H1	*P*. *ridibundus*	–	ON647373
		Veľký lél (SK9)	LSPEES5	D_H4	*P*. *esculentus*	–	ON647388
			LSPEES6	D_H1	*P*. *esculentus*	–	ON647389
			LSPEES8	D_H1	*P*. *esculentus*	–	ON647390
			LSPEES9	D_H1	*P*. *esculentus*	–	ON647391
			LSPEES10	D_H3	*P. esculentus*	–	ON647385
			LSPERI1	D_H1	*P*. *ridibundus*	–	ON647392
			LSPERI6	D_H4	*P*. *ridibundus*	–	ON647393
*Opisthodiscus diplodiscoides*	Slovakia	Chorvátske rameno (SK1)	CHPERI7	O_H1	*P*. *ridibundus*	–	ON647358
		Ivánske jazero (SK4)	IJPERI4	O_H1	*P*. *ridibundus*	–	ON647374
			IJPERI7	O_H1	*P*. *ridibundus*	–	ON647375
		Kráľovská lúka (SK5)	KLPEES2	O_H1	*P*. *esculentus*	–	ON647376
			KLPEES3	O_H1	*P*. *esculentus*	–	ON647377
			KLPERI10	O_H1	*P*. *ridibundus*	–	ON647378
			KLPERI7*	O_H1	*P*. *ridibundus*	ON660890	ON647379
			KLPERI8	O_H1	*P*. *ridibundus*	–	ON647380
		Rusovce (SK6)	RUSPEES3	O_H1	*P*. *esculentus*	–	ON647396
			RUSPEES4	O_H1	*P*. *esculentus*	–	ON647397
			RUSPEES6	O_H1	*P. esculentus*	–	ON647398
			RUSPEES7	O_H1	*P*. *esculentus*	–	ON647399
			RUSPEES1*	O_H3	*P*. *esculentus*	ON660892	ON647394
			RUSPEES2*	O_H1	*P*. *esculentus*	ON660891	ON647395
		Šaštín-Stráže (SK7)	SSPEES2	O_H1	*P*. *esculentus*	–	ON647400
			SSPEES3	O_H2	*P*. *esculentus*	–	ON647401
		Šulianske jazero (SK8)	SULPERI10	O_H1	*P*. *ridibundus*	–	ON647402
			SULPERI4	O_H1	*P*. *ridibundus*	–	ON647403
		Veľký lél (SK9)	LPEES7	O_H4	*P*. *esculentus*	–	ON647381
			LSPEES1	O_H6	*P*. *esculentus*	–	ON647384
			LSPEES2	O_H1	*P*. *esculentus*	–	ON647386
			LSPEES4	O_H5	*P*. *esculentus*	–	ON647387
			LPEES8*	O_H6	*P*. *esculentus*	ON660893	ON647382
			LPEES9*	O_H5	*P*. *esculentus*	ON660894	ON647383

**Fig. 4.**
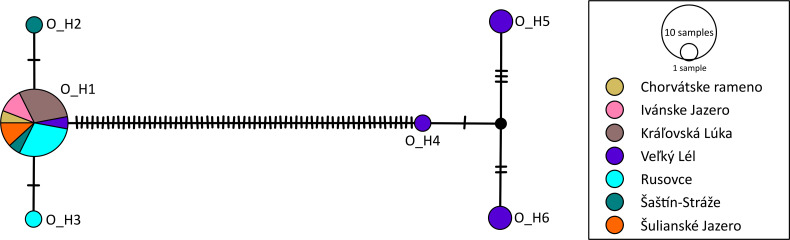
Population-genetic structure of *Opisthodiscus diplodiscoides* found in Slovak populations of water frogs, based on *cox*1 haplotypes presented as a median-joining haplotype network. The sizes of the circles in the network are proportional to the relative frequencies of the haplotypes; small black circles represent missing haplotypes. The vertical lines represent the number of substitutions between individual haplotypes. Different colours represent sample sites according to the legend.

**Fig. 5.**
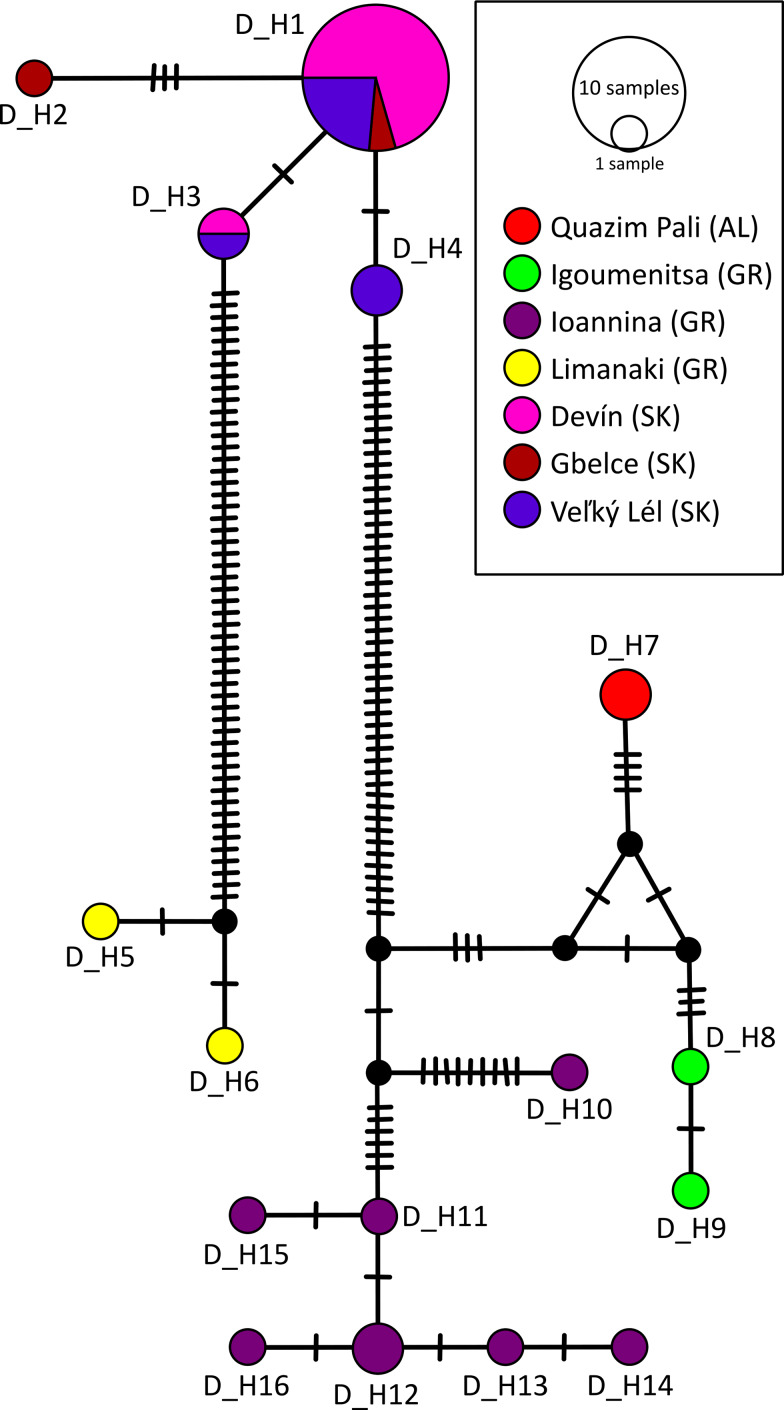
Population-genetic structure of *Diplodiscus subclavatus* based on *cox*1 haplotypes presented as a median-joining haplotype network. The sizes of the circles in the network are proportional to the relative frequencies of the haplotypes; small black circles represent missing haplotypes. Different colours represent sample sites according to the legend. The vertical lines represent the number of substitutions between individual haplotypes. The abbreviations in the legend represent countries where the collection site was situated: AL, Albania; GR, Greece; SK, Slovakia.

Individuals marked by asterisks (*) were sequenced for both *28S* and *cox*1; haplotype = respective haplotype corresponding to Figs 4 and 5 and further discussed in the Results and Discussion section.

## Results

### Taxonomic diversity and the occurrence of water frog paramphistomes in the Balkans and Slovakia

According to morphology, 2 paramphistome species were identified. The first was the diplodiscid *Diplodiscus subclavatus*, which was collected from *P. epeiroticus* and *P. kurtmuelleri* at all 3 Balkan sites, and from *P. esculentus* and *P. ridibundus* at Devín, Gbelce, and Veľký Lél in Slovakia. Our specimens correspond to the morphology of *Diplodiscus subclavatus* originally described by Pallas (1760), mainly by (i) a pyriform body shape with an acetabulum on the posterior end, (ii) a bifurcated caecum reaching the anterior margin of the acetabulum, (iii) a genital pore near the oral opening, (iv) lateral vitelline follicles, (v) a uterus dorsal to testis and (vi) a singular equatorial and median testis at the level of the ovary. The highest prevalence of *D. subclavatus* per host population was reported from *P. esculentus* in Devín (*P* = 80%); however, the highest mean abundance was from *P. epeiroticus* in Ioannina (*a* = 3.80). In localities where *P. ridibundus* and *P. esculentus* occur sympatrically (i.e., Devín and Veľký Lél), *D. subclavatus* individuals were collected from both host species. The second trematode species was the cladorchiid *Opisthodiscus diplodiscoides*, which was present, contrastingly to *D. subclavatus*, in Slovakia but not in the Balkans. The species-specific morphological characteristics of the collected specimens were in accordance with the original description by Cohn (1904), i.e., they had (i) a cylindrical body shape with an acetabulum on the posterior end, (ii) a large acetabulum with an accessory sucker, (iii) a bifurcated caecum terminating at the base of the acetabulum, (iv) a genital opening in front of the bifurcation of the caecum, (v) a uterus occupying the intercaecal field and (vi) 2 testes, preovarian, at the same zone. The overall mean abundance of, and intensity of infection by *O. diplodiscoides* was lower in comparison to *D. subclavatus*, and the highest mean abundance was observed in water frogs from Rusovce and Šulianske jazero (*a* = 0.90). In Veľký Lél, *Opisthodiscus* was collected only from *P. esculentus* but not from sympatrically living *P. ridibundus*. Surprisingly, no mixed infections of both paramphistome species were observed among the investigated host specimens. The quantitative descriptors of parasite populations for each host species per collection site are presented in Table 1.

### Molecular characterization, genetic diversity and phylogenetic relationships of paramphistomes

Sequences of *28S* and *cox*1 from 60 isolates were used to corroborate the taxonomic status of the sampled parasites. Ten and 60 novel sequences of *28S* and *cox*1, respectively, were generated.

The final alignment for assessing the phylogenetic positions of the collected paramphistomes built from *28S* sequences spanned 810 unambiguously aligned nucleotide positions. The ML and BI analyses generated trees with congruent topologies and, therefore, only the BI tree is presented in Fig. 2. The paramphistomes were divided into 2 well-supported phylogenetic lineages, with *Catadiscus marinholutzi* occupying a weakly supported basal position. The first phylogenetic lineage included 5 individuals of *O. diplodiscoides,* preliminary identified according to the morphology – all from water frogs from Slovakia. Weak intraspecific genetic divergence in *28S* was observed among 5 analysed individuals (*p*-distance ⩽0.2%). The second lineage included sequences of all *Diplodiscus* spp. retrieved from GenBank and the novel sequences of 5 individuals of *D. subclavatus*. In comparison to *O. diplodiscoides*, no intraspecific genetic variability in *28S* was observed among *D. subclavatus* individuals. Five isolates of *D. subclavatus* from our study differed from the *D. subclavatus* sequence obtained from GenBank by 6 nucleotide substitutions (*p*-distance = 0.8%), the GenBank sequence, AY222212, being a specimen collected from *P. ridibundus* in Bulgaria.

The second alignment was 683 bp long and included 60 novel isolates of *cox*1. *Zygocotyle lunata* (MT511682) was used as an outgroup for rooting the phylogenetic tree. The ML and BI analyses generated trees with congruent topologies and, therefore, only the BI tree is presented in Fig. 3. Congruently with *28S*, the phylogenetic analyses divided the sequences of all investigated individuals into 2 well-supported clades. Clade 1 included the sequences of all individuals recognized as *O. diplodiscoides*, and 2 phylogenetic lineages were revealed within it. Lineage 1A (PP = 1, BS = 99) encompassed sequences of 19 specimens from all Slovakian collection sites where *O. diplodiscoides* was collected from *P. ridibundus* and *P. esculentus* (*p-*distance ⩽0.3%). In contrast, lineage 1B (PP = 1, BS = 95) included sequences of 5 parasites collected from *P. esculentus* from the site Veľký Lél (SK9). Slightly higher genetic variability was observed among haplotypes from lineage 1B in comparison to haplotypes within lineage 1A (*p-*distance ⩽0.7%). Clade 2 included the sequences of all individuals recognized as *D. subclavatus*, which showed a well-supported sister position to *D. nigromaculati* (PP = 1, BS = 75). Three phylogenetic lineages, with not-fully resolved relationships between them, were recognized within clade 2. Three observed lineages expressed a strong geographic pattern. The first lineage (2A; PP = 0.97, BS = 76) included sequences of 22 *D. subclavatus* individuals collected from *Pelophylax* frogs in Slovakia (*p-*distance ⩽0.6%). The second lineage (2B; PP = 1, BS = 98) encompassed 12 sequences of *D. subclavatus* individuals collected from *P. epeiroticus* in Greece, and *P. kurtmuelleri* in Albania. A notable geographical structure was observed among individuals in lineage 2B, as *D. subclavatus* individuals from *P. kurtmuelleri* in Albania were in the basal position to all Greek individuals, and individuals from Greek sites were divided into 2 lineages according to the collection sites (GR1 and GR2). The last lineage (2C; PP = 1, BS = 98) included the sequences of 2 *D. subclavatus* individuals collected from 1 *P. epeiroticus* individual in southern Greece (Peloponnese Peninsula): collection site GR3 (*p-*distance = 0.3%).

### Population-genetic structure in 2 paramphistome taxa

According to the phylogenetic reconstruction, *cox*1 sequences were divided into 2 lineages: the first one included 24 sequences of *O. diplodiscoides*, and the second one included 36 sequences of *D. subclavatus*. A total of 6 unique *cox*1 haplotypes were recognized in *O. diplodiscoides*, and from 683 nucleotide positions, 71 sites were identified as polymorphic. The overall haplotype diversity (*Hd*) was 0.500, and nucleotide diversity (*π*) reached a value of 3.4%. The majority of the infrapopulations (i.e., parasite individuals from a single host specimen) shared the haplotype O_H1 (Fig. 4). The highest haplotype diversity was observed in individuals from Veľký Lél (site SK9), where, besides 1 individual carrying haplotype O_H1, 5 individuals carried a genetically divergent group of similar haplotypes O_H4-O_H6.

A total of 16 unique *cox*1 haplotypes were recognized in *D. subclavatus*, and from 683 nucleotide positions, 96 sites were identified as polymorphic. The overall haplotype diversity (*Hd*) was 0.778, and nucleotide diversity (*π*) reached a value of 4.5%. Infrapopulations from Slovakia carried 4 similar haplotypes (D_H1-D_H4), and 17 sequences out of 22 collapsed into haplotype D_H1, i.e., this haplotype was identified in individuals from all 3 localities in Slovakia where *Diplodiscus* was present. Sequences from south-western Balkan *Diplodiscus* formed 2 divergent haplogroups. Two similar haplotypes from southern-Greek *Diplodiscus* (Fig. 5, yellow) diverged from all other haplotype clusters. Interestingly, both *Diplodiscus* individuals were collected from a single frog. The other divergent group encompassed haplotypes from northern Greece and Albania, and most of them were represented by singletons. In general, *Diplodiscus* individuals from a single collection site carried genetically similar haplotypes (i.e., only 1 haplotype was recognized among individuals from the site AL1 and 2 similar haplotypes among individuals from the site GR1). The divergent haplotype D_H10 was recognized in 1 *Diplodiscus* individual from Ioannina (ex. 3299); however, it was genetically close to other haplotypes from this collection site (D_H11-D_H16). Similarly to the southern-Greek haplogroup, 2 divergent haplotypes were also recognized in an infrapopulations from 2 frogs (ex. 3609a – D_H11 and ex. 3609b – D_H14 and ex. 3614a – D_H13 and ex. 3614b – D_H12), suggesting repetitive infections of a single host by *Diplodiscus*.

## Discussion

Helminth communities in *Pelophylax* water frogs have been studied relatively extensively but most studies have employed only a basic morphological approach for species determination (e.g., Vojtková and Roca, 1994, 1996; Bjelić-Čabrilo, *et al*., 2009; Popiołek *et al*., 2011; Okulewicz *et al*., 2014; Herczeg *et al*., 2016; Kuzmin *et al*., 2020). They were not focused on genetic diversity, and therefore might have underestimated the real species diversity of frog parasites. According to Carlson *et al*. (2020), the helminth fauna of amphibians appears to be strongly underexplored in comparison to other vertebrate taxa, and even in Europe, we can find some regions, such as the Balkan Peninsula, which are still only superficially investigated in this regard. In the present study, we therefore focused on these neglected regions. By applying molecular methods, we revealed unexpected genetic variability in the mitochondrial *cox*1 fragment for diplodiscids, exhibiting also a geographic structure associated with the distribution of the *Pelophylax* frog hosts.

Our study revealed the presence of 2 paramphistome taxa in Slovak water frogs. The first was identified as *Opisthodiscus diplodiscoides*, with average to low prevalence per host population and low intensity of infection per examined host individual, and the second was *Diplodiscus subclavatus*, with comparatively higher prevalence and infection intensity. Previously, representatives of the genus *Opisthodiscus* have been found relatively rarely in European amphibians, in comparison to those of the genus *Diplodiscus* (Bailenger and Chanseau, 1954; Vojtková *et al*., 1963; Combes and Gerbaux, 1970; Combes and Sarrouy, 1971; Grabda-Kazubska, 1980; Martínez-Fernández *et al*., 1988; Vojtková and Roca, 1994; Andreas, 2007; Navarro and Lluch, 2006). In Western Europe, the genus *Opisthodiscus* is represented by *O. nigrivasis* parasitizing endemic Iberian *P. perezi* (López-Seoane, 1885) and widely distributed *P. esculentus* (Combes and Gerbaux, 1970; Combes and Sarrouy, 1971; Martínez-Fernández *et al*., 1988). In addition, Rodrigues (2014) documented *O. nigrivasis* also from non-native *Xenopus laevis* (Daudin, 1802) in Portugal, indicating its strong host switch potential. In central and eastern Europe, the more common species is *O. diplodiscoides*, which was almost exclusively reported from *P. esculentus* or *P. ridibundus* (Vojtková, 1974; Grabda-Kazubska, 1980; Andreas, 2007). Nevertheless, the identification of the 2 *Opisthodiscus* species is often unreliable in older literature, and, therefore, the real distribution ranges are uncertain and might overlap (Odening, 1959). Vojtková *et al*. (1963) reported rare infections by *O. diplodiscoides* in *P. esculentus* in southern Slovakia (around city Komárno), which is congruent with our observations. We detected this parasite in *P. ridibundus* and *P. esculentus* frogs from all investigated sites in the Pannonian Basin. Most of them were geographically close to the sites from Vojtková *et al*. (1963) (SK1, SK4-SK6, SK8 and SK9), with only 1 site (SK7) situated further, in the north-western corner of the Pannonian Basin. Surprisingly, only in Veľký Lél (SK9) was *O. diplodiscoides* present in the host population also with *D. subclavatus*. However, in 20 examined *P. esculentus* individuals, we did not observe any instances of coinfection by both species, which might indicate that *D. subclavatus* and *O. diplodiscoides* exhibit antagonistic relationships. Interspecific competition between parasite taxa has been widely studied among adult helminths in their definitive vertebrate hosts (see Poulin, 2007 and references therein) and was also documented among anuran parasites sharing microhabitats within the host, i.e., between *Rhabdias bufonis* (Schrank, 1788) Stiles and Hassall, 1905 (Nematoda), *Haematoloechus variegatus* (Rudolphi, 1819) and *Haplometra cylindracea* (Zeder, 1800) (Trematoda) parasitizing the lungs of frogs (Mazurmovič, 1957; Okulewicz *et al*., 2014), and between *Aplectana acuminata* (Schrank, 1788) (Nematoda) and *Opisthioglyphe ranae* (Fröhlich, 1791) (Trematoda), which are intestinal parasites (Mazurmovič, 1957; Bjelić-Čabrilo *et al*., 2009). The presence of 1 parasite species in the host might preclude other parasites from becoming established (Plasota, 1969; Bjelić-Čabrilo *et al*., 2009). A similar situation might be hypothesized in the case of *Diplodiscus* and *Opisthodiscus* where both share the same microhabitat within the frog, i.e., the rectum and the terminal end of the intestine. An antagonistic relationship was suggested for these species also by Navarro and Lluch (2006), who similarly reported no mixed infection by *D. subclavatus* and *O. nigrivasis* in Moroccan *P. saharicus* (Boulenger, 1913). Unfortunately, interspecies relationships were not thoroughly investigated in the present study, and the limited host sample size per collection site, in combination with the relatively low prevalence of the 2 potentially antagonistic species, impede the drawing of complex conclusions. Thus, further research is necessary to reveal whether the antagonistic relationship is due to competitive resource exploitation (Mouritsen and Andersen, 2017), the active elimination of a coexisting competitor (e.g., predation, Polis *et al*., 1989), or the passive release of metabolites negatively impacting the competitor (i.e., interference competition, see review by Mideo, 2009).

The phylogenetic analyses suggested that the investigated *Opisthodiscus* individuals were divided into 2 phylogenetic lineages. The genetic divergence was supported by both molecular markers, and while the first phylogenetic group encompassed individuals from all collection sites where the presence of *Opisthodiscus* was documented, the second group included only 5 individuals collected from *P. esculentus* at the Veľký Lél site (SK9). Genetic variability was even more prominent in the mitochondrial *cox*1 gene, as 3 divergent haplotypes (O_H4-O_H6) were recognized in these 5 *Opisthodiscus* individuals. Such observations might indicate either the presence of 2 *Opisthodiscus* taxa in Southern Slovakia (e.g., *O. diplodiscoides* and *O. nigrivasis*, or *O. diplodiscoides* and another taxon) or substantial intraspecific differentiation within *O. diplodiscoides*. Unfortunately, a morphological evaluation that might have shed light on the taxonomic status of genetically diverged *O. diplodiscoides* individuals was not possible, as all 4 specimens collected from infrapopulations carrying haplotypes O_H1-O_H3 were either highly damaged (2 individuals), and therefore immeasurable, or did not show any significant differences in comparison to *O. diplodiscoides* individuals from infrapopulations carrying the divergent haplotypes O_H1-O_H3 (2 individuals). Such strong genetic differentiation might be due to the geographically distant origin of some *Opisthodiscus* individuals, potentially introduced with intermediate hosts (i.e., aquatic snails) or definitive hosts (i.e., amphibians). Information about the life cycle of *Opisthodiscus* is scarce; however, Simón-Vicente *et al*. (1974) reported *Ancylus fluviatilis* (O. F. Müller, 1774) as the intermediate host for *O. nigrivasis,* a gastropod with a distribution range covering almost the entirety of Europe, including Slovakia (Cordellier and Pfenninger, 2008; Horsák *et al*., 2021). Freshwater molluscs exhibit various upstream and downstream dispersal capabilities which can be even further increased by anthropogenic vectors (Kappes and Haase, 2012). Since the water bodies in Veľký Lél are river arms and gravel pits with direct communication with the Danube River, the Danube might represent an optimal pathway for the transmission of gastropods (including *A. fluviatilis*), thus introducing divergent genetic variants of larval stages of trematodes into geographically distant regions (e.g., *O. diplodiscoides*).

*Diplodiscus subclavatus* was collected from all 4 investigated *Pelophylax* species across both geographical regions, and exhibited a higher host range, and also genetic diversity, in comparison with the *O. diplodiscoides*. *Diplodiscus* species appear to be common parasites of *Pelophylax* frogs across the distribution range of the parasite genus, i.e., from east Asia (Men *et al*., 2016) up to the western Mediterranean region (Navarro and Lluch, 2006). In Europe, the only species of the genus is *D. subclavatus* (e.g., Bailenger and Chanseau, 1954; Vojtková and Roca, 1994; Andreas, 2007; Okulewicz *et al*., 2014; Herczeg *et al*., 2016; Kuzmin *et al*., 2020), which was also documented in our study. The wide host range of this parasite species, exhibiting a life cycle with the planorbid snail as an intermediate host (e.g., *Planorbis planorbis* (Linnaeus, 1758), *P. umbilicalis* Benson, 1836, *Anisus vortex* (Linnaeus, 1758) or *Gyraulus albus* (O. F. Müller, 1774), Aho (1990); Cichy *et al*. (2011)) and frogs as definitive hosts, suggests its low degree of host specificity, and therefore might explain its wide distribution range. Nevertheless, the genetic variability within the species was not previously studied.

The herein used mitochondrial genetic markers revealed exceptionally high genetic variability in *cox*1, although the more conservative *28S* genetic marker did not show any variability among the investigated individuals. Both, the phylogenetic tree and the haplotype network, divided *cox*1 sequences into 3 divergent haplogroups. The relationships between haplogroups were not fully resolved in the phylogenetic tree; however, the network suggested that 2 Balkan haplogroups had a closer relationship to the haplogroup from Slovak *D. subclavatus* than to each other. Nonetheless, this might be an artefact of the genetic distances between the 3 haplogroups being too high, resulting in, at first sight, an illogical pairing in the network. In contrast to Slovak *D. subclavatus*, where no genetic structure was observed regarding the geographical distribution (i.e., the majority of infrapopulations carried the same haplotype), a spatial genetic structure was apparent in the Balkans - individuals from the same collection site carried similar haplotypes. Furthermore, in comparison to infrapopulations from Slovakia, the observed haplotype diversity was much higher in the Balkans. High intraspecific genetic diversity in southern Europe compared to the more northerly areas has been observed in many organisms and is associated with a long-term allopatric divergence in refugial populations during the Pleistocene glacials (e.g., Taberlet *et al*., 1998; Hewitt, 2000, 2004). Alternatively, high genetic diversity might also be a result of the current gene flow between adjacent populations that are not fully geographically isolated. The first assumption would be in congruence with the proposed phylogeographical scenario for *Pelophylax* frogs and their assumed historical dispersion. Molecular clocks indicate that the basal radiation within western Palearctic water frogs took place in the middle Miocene, with subsequent speciation events during the upper Miocene and the Pliocene (Lymberakis *et al*., 2007; Wiens *et al*., 2009; Akın *et al*., 2010). Later, during the Pleistocene, it is supposed that water frogs (and also other organisms) expanded to the north during the interglacial periods and retracted back to the south in the glacials (Snell *et al*., 2005; Hoffmann *et al*., 2015). During the ice ages, these southern regions served as refugia which were often divided into several microrefugia (Schmitt, 2007; Schmitt and Varga, 2012). As the distribution and dispersion of parasites are strongly limited and dependent on the dispersal capabilities of their hosts (Blouin *et al*., 1995), it is tempting to assume that a similar distribution pattern will be observed also for the parasites of *Pelophylax* frogs.

The existence of microrefugia in the Balkans might also explain the existence of 2 diverse haplogroups among the Balkan *Diplodiscus*. Recent studies indicate that the Peloponnese Peninsula might have represented such a microrefugium for several taxa (e.g., *Mesobuthus* scorpions (Parmakelis *et al*., 2006); *Parnassius* butterflies (Gratton *et al*., 2008); *Anguis* legless lizards (Jablonski *et al*., 2016); or *Pelobates* frogs (Dufresnes *et al*., 2019). Moreover, Radojičić *et al*. (2015) observed genetic divergence between *P. epeiroticus* populations in Northern and Southern Greece, further supporting our assumption of the divergence of *Pelophylax* frogs with *Diplodiscus*.

Congruently with our observations, from the genus *Diplodiscus* only *D. subclavatus* was previously reported from Slovakia (Vojtková *et al*., 1963; Vojtková, 1974) and the Balkans (Vojtková and Roca, 1994). Surprisingly, the phylogenetic reconstruction based on *28S* and the computation of pair-wise genetic distances revealed quite substantial genetic differences between *D. subclavatus* in our study and the sequence used as a representative of the species which was employed in phylogeny by Olson *et al*. (2003) (AY222212).

Although the observed high genetic variability might suggest the existence of 3 divergent *Diplodiscus* taxa in the studied regions – 2 in the Balkans and 1 in Slovakia – the morphological data (i.e., morphometrics) did not reveal any statistically significant differences between individuals from the respective parasite metapopulations. However, due to the insufficient quality of the material, and its scarcity, we couldn't obtain a comparable dataset from each suggested group, and, therefore, further collection and morphological re-evaluation are necessary to confirm the taxonomical status of the 3 genetic haplogroups.

The phylogenetic relationships within the family Diplodiscidae were first tackled by Sey (1983), who, according to the morphological apomorphies and geographical distribution, split diplodiscids into 2 lineages – Opisthodiscinae, encompassing *Opisthodiscus* and *Megalodiscus*, and Diplodiscinae, encompassing all other Diplodiscidae genera. Later, Sey (1988, 1991) re-classified *Opisthodiscus* and *Megalodiscus* into Megalodiscinae within Cladorchiidae; however, this taxonomical revision was not generally accepted. Our reconstruction based on the conservative *28S* gene did not support the monophyly of the family Cladorchiidae (*sensu* Alves *et al*., 2020), although it was previously suggested (nonetheless, only on the basis of weak support). Even though *Opisthodiscus* and *Megalodiscus* share similar morphological traits (e.g., 2 testes, and an oesophagus that is shorter than the pharynx, Sey, 1991), recent phylogenetic analyses based on molecular data (Alves *et al*., 2020; Queiroz *et al*., 2021; and this study) suggest that these 2 genera are in paraphyly, and while the position of *Opisthodiscus* is nested within Diplodiscidae (i.e., a sister position to species of the genus *Diplodiscus*), *Megalodiscus* is positioned within Cladorchiidae. Thus, we can only assume that convergent adaptation to amphibian hosts evolved among paramphistomoids in different geographical regions; however, to fully resolve the classification of cladorchiids, more comprehensive morphological evaluation is required. The sister position (albeit weakly supported) of South American *Catadiscus* to Eurasian *Opisthodiscus* and *Diplodiscus* with a deep nodal split might indicate historical allopatric divergence during the ancient division of the continents (as proposed by Sey, 1983). Although, the monophyly of Diplodiscidae was not fully supported by Queiroz *et al*. (2021), the current addition of *Opisthodiscus* into phylogeny helps to shed more light on the relationships within this family. Moreover, with further addition of (still missing) molecular data from other genera from the family Diplodiscidae (i.e., *Progonimodiscus* Vercammen-Grandjean, 1960 (in frogs from Africa), *Dermatemytrema* Price, 1937 (in turtles from North America), *Pseudodiplodiscus* Manter, 1962 (in fish from South America) and *Australodiscus* Sey, 1983 (in frogs from Oceania – Australia) (Sey, 1991; Jones, 2005)) might help to finally resolve relationships of the diplodiscids to the cladorchiids, and further elucidate the historical dispersion routes and diversification within diplodiscids.
